# Resolution of complex ends by Nonhomologous end joining - better to be lucky than good?

**DOI:** 10.1186/2041-9414-3-10

**Published:** 2012-12-31

**Authors:** Natasha Tiffany Strande, Crystal Ann Waters, Dale A Ramsden

**Affiliations:** 1 Lineberger Comprehensive Cancer Center, Department of Biochemistry and Biophysics and Curriculum in Genetics and Molecular Biology, University of North Carolina, Chapel Hill, North Carolina 27599, USA

**Keywords:** Double strand break repair, Nonhomologous end joining, DNA damage, Ionizing radiation

## Abstract

The Nonhomologous end joining pathway is essential for efficient repair of chromosome double strand breaks. This pathway consequently plays a key role in cellular resistance to break-inducing exogenous agents, as well as in the developmentally-programmed recombinations that are required for adaptive immunity. Chromosome breaks often have complex or “dirty” end structures that can interfere with the critical ligation step in this pathway; we review here how Nonhomologous end joining resolves such breaks.

## Double strand break repair and complex end structures

DNA double strand breaks (DSBs) arise after replication, aberrant repair of spontaneous damage, and exposure to exogenous damaging agents, especially those used in cancer therapies. DSBs are also intermediates in several developmentally-programmed recombinations. Failed DSB repair is typically lethal, while aberrant DSB repair can lead to developmental defects, progeria, and cancer. Repair pathways include Homologous recombination (HR), Nonhomologous end joining (NHEJ), and Alternate end joining (Alt-EJ) (reviewed in e.g. [1]). Importantly, HR is dependent on extensive (100s to 1000s of nucleotides) DNA synthesis, a sister chromatid template to direct this synthesis, and a homology search step needed to find the template in a sister chromatid. In contrast, NHEJ is primarily a ligation reaction and can act independently of S-phase restricted sister chromatids, dNTP generation [2], and other requirements for extensive synthesis. Finally, a fraction of ligation-mediated repair is independent of factors required for classically defined NHEJ, and is thus termed “Alt-EJ”.

**Figure 1:**
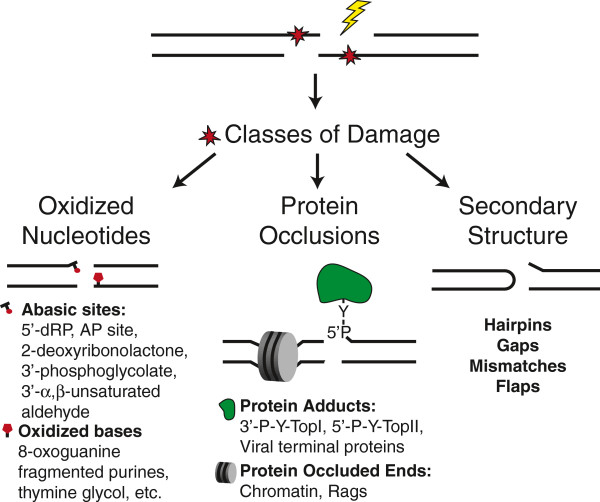
**Biological sources of DSBs generate complex end structures. **Examples include oxidized nucleotides, protein occlusions, and secondary structures.

The primary disadvantage to resolving ends by ligation is that biological sources of DSBs often produce “dirty” or complex end structures that can interfere with this step (Figure 1). DNA flanking the break may possess nucleotide damage, most frequently oxidized bases, various classes of abasic sites, and 3^′^ phosphate or 5^′^ hydroxyl termini [3]. Such damage is especially likely in the case of ionizing radiation-induced breaks, which are associated with damage clusters [3,4]. DSB ends may be further occluded by proteins, both non-covalently associated (e.g. chromatin) and covalently adducted type II topoisomerases [5]. Ends can also possess secondary structures including hairpins or, after a pair of ends are aligned together, gaps, mismatches, or flaps.

**Figure 2:**
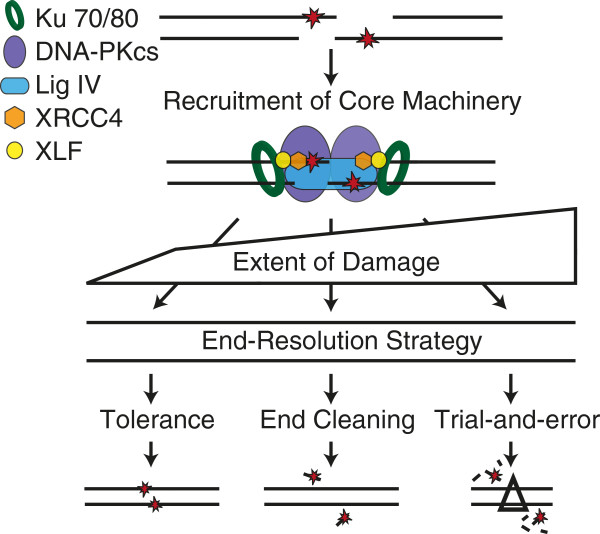
**NHEJ end-resolution strategies. **Resolution of complex ends by NHEJ first requires the recruitment of the core machinery (Ku, DNA-PKcs, Ligase IV, XRCC4, and XLF). The type and extent of damage varies, and this in turn probably dictates choice of strategy.

**Figure 3:**
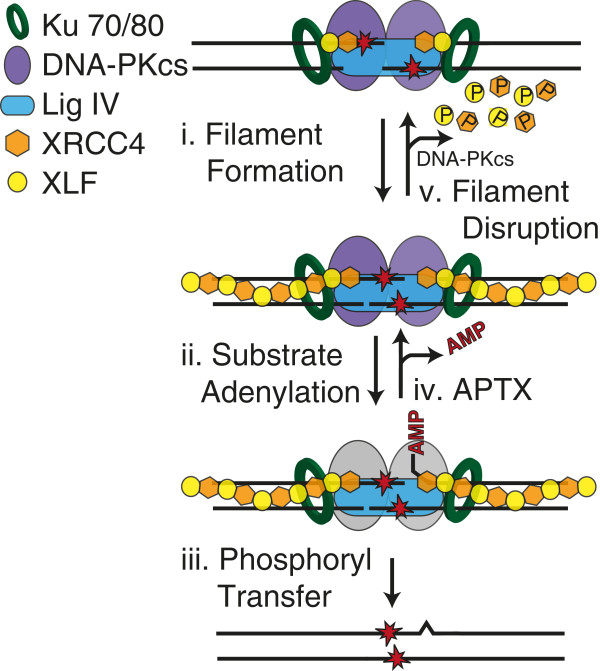
**Joining by tolerance. **The NHEJ core machinery can be associated with an XLF-XRCC4 filament that may stabilize aligned complex end structures (i) sufficiently to support transfer of the adenylate from the ligase to 5^′ ^phosphate terminus (ii), possibly through to complete ligation (iii). If ligation aborts, the 5^′^ adenylated (AMP) intermediate can be removed by Aprataxin (APTX) (iv). DNA-PKcs-directed phosphorylation of XLF and XRCC4 can disrupt the XLF-XRCC4 filament (v) to allow processing factors access to the DNA to remove the obstruction.

**Figure 4:**
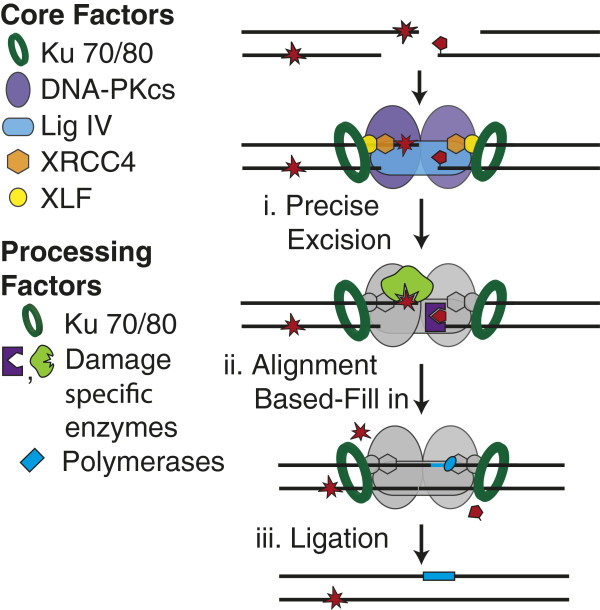
**End cleaning. **Ligation-blocking damage can be excised by enzymes (e.g. APTX, PNKP, APLF, Tdps, Ku; purple and green proteins) that recognize and remove specific end structures (i). Family X polymerases (light blue oval) can replace the excised DNA (ii) prior to ligation (iii).

**Figure 5:**
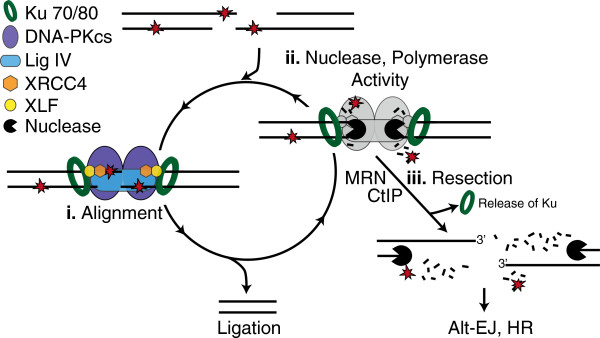
**Trial and error. **Ends can go through sequential rounds of end alignment (i) and processing (ii) (primarily nucleases; black Pac-man) until ligated. Alternatively, ends can be resected (iii) and Ku released, enabling resolution of ends by homologous recombination (HR) or alternative end-joining (Alt-EJ) pathways.

NHEJ resolves complex ends by employing a sophisticated machine engineered to facilitate ligation despite ligation-blocking lesions. A series of core factors are necessary and sufficient to recognize ends, align a pair of ends together, and perform the ligation step (Figure 2). Core factors include 1) Ku; a DNA end binding heterodimer of 70 and 80 kD subunits (Ku70, Ku80), 2) XRCC4-ligase IV (X4-LIV); an obligate oligomer of a ligase catalytic subunit (DNA ligase IV) and scaffolding subunit (XRCC4), 3) XRCC4-like factor (XLF, also termed Cernunnos), and 4) DNA dependent protein kinase catalytic subunit (DNA-PKcs); a 450 kD kinase recruited to ends by Ku (reviewed in [6]). However, additional factors are required, both to integrate NHEJ with the DNA damage response and local chromatin structure [7], as well as (to be discussed here) to help this core machine resolve complex ends (Table 1). NHEJ employs these additional factors according to strategies (Figure 2) for resolving complex ends we suggest can be roughly categorized as 1) tolerance (Figure 3), 2) end “cleaning” (Figure 4), and 3) trial-and-error (Figure 5).

**Table 1:** End processing factors

Factor*	**Activity**
APTX	Removes 5^′^-adenylate adducts [40]
PNKP	Removes 3^′^ phosphates and phosphorylates 5^′^ hydroxyls [45]
APLF	Histone chaperone [59] 3^′^-5^′^ exonuclease, endonuclease [56,61]
TDP1	Removes Top I adducts [63], 3^′^ deoxyribose fragments [47,67,68]
TDP2	Removes Top II adducts [64]
XRCC5,XRCC6 (Ku)	Removes 5^′^-dRP residues and abasic sites [27]
POLM (Pol *λ*)	Fills in gaps when ends align with no complementarity [90]
POLL (Pol μ)	Fills in gaps when ends are partly complementary [86,90]
DCLRE1C (Artemis)	Endonuclease, 5^′^-3^′^ exonuclease [100]
WRN	3^′^-5^′^ exonuclease [121,122] and 3^′^-5^′^ helicase [120]
MRE11/RAD50/NBN (MRN)	3^′^-5^′^ exonuclease, endonuclease [101,129]
SETMAR (Metnase)	Endonuclease/exonuclease [103]

*HUGO gene nomenclature: *APTX*, aprataxin; *PNKP*, polynucleotide kinase 3^′^-phosphatase; *APLF*, aprataxin and PNKP like factor; *TDP1*, tyrosyl-DNA phosphodiesterase 1; *TDP2*, tyrosyl-DNA phosphodiesterase 2; *XRCC5*,*XRCC6 (Ku80, Ku70)*, X-ray repair complementing defective repair in Chinese hamster cells 5/6; *POLM*, polymerase mu; *POLL*, polymerase lambda; *DCLRE1C (Artemis)*, DNA cross-link repair 1C; *WRN*, Werner syndrome; *MRE11/RAD50/NBN*, meiotic recombination 11 homolog/RAD50 homolog/nibrin (Nbs1), SETMAR, SET domain and mariner transposase fusion gene (Metnase).

## Tolerance of complex ends by low fidelity ligation

Ligation initiates with the transfer of an adenosine monophosphate from a lysine within the ligase active site to the 5^′^ phosphate (Figure 3, step ii). The final step in ligation has similarities to DNA synthesis, as the new phosphodiester bond is made by a phosphoryl transfer reaction, where the 3^′^ hydroxyl terminus of one strand performs a nucleophillic attack on the activated 5^′^ phosphate terminus of the second (Figure 3, step iii) (reviewed in [8]). Importantly, most DNA ligases resemble polymerases in that they are most active when joining strands with termini complementary to a “template” strand. Thus, in the same manner as polymerases, ligases can be considered as high or low fidelity according to the degree to which they tolerate mispairs or other helix distortions in DNA flanking the bond to be made.

### The XLF-X4-LIV splint and low fidelity ligation

X4-LIV can join together strand termini with flanking mispairs more readily than other ligases [9,10], and thus can be considered to be of low fidelity. Importantly, XLF specifically promotes low-fidelity ligation [11,12]. Dimers of XLF interact with dimers of XRCC4 and DNA [13-18], resulting in DNA- bound (XLF-XRCC4)_N_ filaments [14,19-23] that eventually include or terminate in a X4-LIV complex (Figure 3, step i). These filaments are thought to act as a protein-“splint”, stabilizing an aligned pair of ends. The splint could account for reduced ligation fidelity by suppressing helix distortions associated after alignment of complex end structures, or by simply increasing the time the active site has to work with end alignments with poorly oriented termini. Low-fidelity ligation is advantageous for NHEJ as it increases the spectrum of complex ends that can be ligated together directly without prior end processing.

However, there is a limit to what NHEJ’s ligase can tolerate [9,24-30], even with the XLF-X4-LIV splint. Notably, DNA-PKcs-mediated phosphorylation of multiple sites within XLF and XRCC4 disrupts the filament (Figure 3, step v) [31]. This may relax (or release) unproductive complexes of the ligase and aligned ends to give better access to end processing enzymes required for other NHEJ strategies (e.g, Figures 4, 5).

Additionally, like damage tolerance by translesion polymerases in replication, tolerance of complex end structures by NHEJ is a form of procrastination – mismatched and damaged nucleotides flanking the break site are not repaired and thus retained in the joined product (junction). Unresolved damage will interfere with subsequent transcription or replication through the junction. Additionally, attempted repair by other pathways (e.g. base excision repair; BER) of residual damage clusters in junctions risks re-breaking the site [32-36]. Sustained localization of NHEJ factors (e.g. Ku, X4-LIV, XLF) after joining may even help regulate BER activity.

## Damage-specific end cleaning

Ligases cannot join ends unless they have 5^′^ phosphate and 3^′^ hydroxyl termini, regardless of how stably a pair of aligned termini are juxtaposed. In addition, NHEJ’s ligation step is blocked by terminal or near-terminal abasic sites [24-27,29] and when lesions or mismatches in end structures are sufficiently helix distorting. Common terminus-blocking lesions can be excised and the potentially resulting gaps filled in before ligation (Figure 4). As with other repair pathways (e.g. BER), NHEJ can thus fully restore sequence at DSB sites, even when the break was associated with ligation blocking damage [24].

To this end, NHEJ employs an array of enzymes that partly overlap with BER and single strand break repair (SSBR). Indeed, three of these factors – aprataxin (APTX), polynucleotide kinase/phosphatase (PNKP), and aprataxin and polynucleotide kinase/phosphatase like factor (APLF) - employ N-terminal forkhead associated (FHA) domains to mediate their participation in both NHEJ and BER/SSBR pathways (reviewed in [37]). These domains physically interact with XRCC4 [38] and XRCC1 [39] to direct their participation in NHEJ and BER/SSBR, respectively. FHA domain-mediated interactions are stimulated by phosphorylation of XRCC4 and XRCC1 by casein kinase II.

### Aprataxin

As noted above, there is a limit to what the ligase can tolerate. Attempts to tolerate complex end structures can result in ligation failure at an intermediate step, after adenylation of the 5^′^ terminus but before the final phosphoryl transfer (Figure 3, step ii). A new ligase IV molecule cannot act on the 5^′^ adenylated product of aborted ligation; Aprataxin (APTX) resets the substrate for another ligation attempt by removing 5^′^-adenylate adducts (Figure 3, step iv), as mediated by APTX’s zinc-finger-histidine triad (HIT) domain [40].

Mutations in APTX account for several neurodegenerative disorders, including ataxia with oculomotor apraxia type 1 (AOA1) [41,42]. However, sensitivity of APTX deficient cells to various DNA damaging agents is mild [43], and it has been difficult to detect measurable differences in either SSBR or DSBR ([44] and references therein). APTX is thus argued to act on a minor subset of breaks [44], with the consequences of failed action in an organism possibly disproportionate to the low frequency of these events.

### Polynucleotide kinase/phosphatase

5^′^ hydroxyl and 3^′^ phosphate termini are generated directly by reactive oxygen species, after metabolization of certain strand breaks (3^′^- phosphoglycolate, 3^′^-phosphotyrosine), or by the action of endo-VIII-like glycosylases (Neil1 or Neil2). PNKP has two catalytic domains sufficient to prepare such termini for ligation: a central domain that removes 3^′^ phosphates, and a C-terminal domain that phosphorylates 5^′^ hydroxyls [45] (reviewed in [46]). PNKP was also shown to act coordinately with TDP1 to remove the 3^′^ phosphate generated after removal of 3^′^-phosphoglycolate residues [47].

Mutations in PNKP result in microcephaly with early onset intractable seizures and developmental delay (MCSZ) [48]. A role for PNKP specifically in double strand break repair is supported by sensitivity of PNKP deficient cells to ionizing radiation [48,49], and PNKP is required for NHEJ of ends without 5^′^ phosphate in cell extracts [50]. PNKP’s contribution to radiation sensitivity relies to some extent on damage-dependent phosphorylation of sites in PNKP by DNA-PKcs and ATM [51,52]*.*

### Aprataxin and polynucleotide kinase/phosphatase like factor

APLF (also referred to as PALF, C2orf13, and Xip1) possesses a tandem pair of poly(ADP-ribose) binding zinc finger (PBZ) motifs [53], which mediate recruitment of APLF to damage [54-57] after poly(ADP) ribose polymerase-3 (PARP-3) modification of flanking chromatin [58]. Disruption of APLF’s PBZ domains attenuates X4-LIV accumulation at DSB ends in cells, which in turn results in defects in NHEJ of radiation induced breaks and DSB intermediates in class switch recombination [58]. This may be at least partly because a network of interactions between APLF, Ku [56,57], X4-LIV [57,58], DNA, and poly(ADP-ribose) chromatin [57,58] could be required for stable assembly of an NHEJ complex at ends. APLF also possesses a conserved C-terminal domain with a NAP1L family histone chaperone motif, which is sufficient to promote assembly and disassembly of nucleosomes and nucleosome substructures *in vitro*[59]. Ku can recognize DSB ends even when on the surface of a nucleosome [28], but an active NHEJ complex, including X4-LIV and DNA-PKcs, requires at least 60 bp of free DNA flanking the end [60] (probably more with an (XLF-X4)_N_ splint). APLF, perhaps triggered by coordinate recognition of ends by Ku and PBZ mediated interactions with flanking poly(ADP-ribose) modified chromatin, could direct a very limited remodeling of nucleosomes flanking broken ends to make room for subsequent loading of X4-LIV. APLF may additionally act as an exonuclease and structure-specific endonuclease [56,61] to resolve mismatches and flaps.

### Tyrosyl DNA phosphodiesterases

Topoisomerases (Top I, Top II) resolve DNA topological stress associated with replication and transcription. They employ a cleavage/ligation cycle with an intermediate step where a tyrosine in the topoisomerase is covalently linked to a strand break through 3^′^ or 5^′^ phosphate termini (Top I and top II, respectively) [62]. The cleavage/ligation equilibrium can be altered (e.g. after treatment with topoisomerase poisons) such that cells accumulate strand breaks with termini adducted to a topoisomerase through their active site tyrosine [5]. If a type I topoisomerase aborts, the topoisomerase is adducted to a single strand break, 3^′^ phosphate terminus; if a type II topoisomerase aborts, the topoisomerase is adducted to DSB 5^′^ phosphate termini. Adducted topoisomerases can be removed from DNA strand termini by reversal of the covalent intermediate, through the action of tyrosyl-DNA phosphodiesterases Tdp1 [63] and Tdp2 [64].

Tdp1’s primary substrates are the 3^′^-phosphotyrosine adducts generated by aborted type I topoisomerase activity, either at SSBs, or DSBs generated after replication through unrepaired SSBs [65,66]. Epistasis analysis indicates Tdp1 activity on even the DSB-associated products is upstream of repair by HR in *S. cerevisae*[66]. However, Tdp1 additionally has activity on other 3^′^ phosphate adducts including 3^′^-phosphoglycolates [67-71] (Table 1), the most common class of nucleotide damage associated with ionizing radiation induced strand breaks. Tdp1 is required for resolution by NHEJ of substrates with 3^′^-phosphoglycolate termini in a cell extract model [47], and patients with Tdp1 mutations (Spinocerebellar ataxia with axonal neuropathy 1; SCAN-1) [72,73] are sensitive to radiomimetic drugs that can introduce strand breaks with 3^′^-phosphoglycolate termini [47,74]. At the same time there are significant backup pathways active in cells [75], possibly explaining why SCAN1 cells are neither severely sensitive to ionizing radiation [71], nor possess obvious defects in rates of DSB repair after ionizing radiation [76].

Tdp2 (also termed TTRAP; TRAF and TNF receptor-associated protein, and EAPII; ETS1 associated protein II) is most active in removing tyrosines adducted to 5^′^ phosphates at DSB ends [64,77], a product of aborted type II topoisomerase action. Consistent with this specificity, Tdp2 is essential for resistance of chicken DT-40 cells to type II topoisomerase poisons (e.g. etoposide) [78]. Tdp2 could in principle participate in either HR or NHEJ pathways for DSB repair, and there are as yet no reported epistasis analyses or physical interactions specifically linking Tdp2 to either. Nevertheless, a role for Tdp2 uniquely within NHEJ seems most likely. Unlike Top I adducts, there is little use for “clean” removal of Top II adducts within the HR pathway, since the 5^′^ strand must anyway be extensively resected as a pre-requisite for the homology search step. Indeed, removal of the Top II-like Spo11 adduct at DSB intermediates during meiotic HR relies on the Mre11/Nbs1/CtIP complex; this latter pathway is apparently Tdp2 independent, as the excised Spo11 is still adducted to a short oligonucleotide [79].

### Ku

DSB with associated abasic sites, either 5^′^ terminal (5^′^-deoxyribose phosphate; 5^′^-dRP) or near-terminal (apurinic/apyrimidinic; AP), can be generated directly by ionizing radiation. However, they are probably more frequently associated with DSB products of aborted base excision repair, including the DSB intermediates in immunoglobulin class switch recombination [80,81]. Regardless of source, NHEJ cannot join such ends together both *in vitro* (whether or not XLF is present) [25-27,29] or in cells [27,29] unless the abasic site is excised. Excision of these abasic sites is mediated both *in vitro* and in cells primarily by the Ku heterodimer which, in addition to its primary role in recognizing ends and recruiting other factors, is a 5^′^-dRP/AP lyase [27]. Notably, Ku’s 5^′^-dRP/AP activity is primarily restricted to substrates where incision is both necessary and sufficient to prepare ends for the ligation step [29]. Specifically, Ku is much less active on abasic sites near 3^′^ termini, where incision by a lyase would leave a ligation blocking 3^′^-α, β-unsaturated aldehyde. Ku is similarly much less active when abasic sites near 5^′^ termini are significantly embedded in duplex DNA, a context where the abasic site no longer blocks the ligation step. The latter substrate specificity is essentially non-overlapping relative to abasic site metabolizing enzymes implicated in BER (AP endonuclease, pol β), whose activities are mostly restricted to sites with significant (>4 bp) flanking dsDNA [26,27,29].

### Family X polymerases

A variety of polymerases have been implicated in NHEJ (also reviewed in [82]), but the majority of evidence favors a primary role for several members of the mammalian family X polymerase: Pol *λ*, Pol μ, and Terminal deoxynucleotidyl transferase (TdT). All three polymerases possess homologous N-terminal BRCT (Breast cancer associated carboxy-terminal) domains [83] that promote formation of a complex including the polymerase, Ku and X4-LIV at DNA ends [84-90]. BRCT domains have no impact on intrinsic catalytic activity but are essential for the participation of the polymerases in NHEJ, emphasizing the importance of coupling their catalytic activities to a complex of aligned ends [85,87,90]. The three polymerases have distinct substrate requirements and activities, with decreasing dependence on template strand, in order Pol *λ*>Pol μ>TdT [90]. All three have been clearly implicated in repair by NHEJ of intermediates in V(D)J recombination [91-94], with TdT’s entirely template independent activity observed only during V(D)J recombination by virtue of its restricted expression [95]. The other two polymerases are expressed in all cell types and have overlapping activities [83,96], making it difficult to parse their relative contributions. In general, they fill in gaps present after alignment of broken ends (the gap typically a consequence of prior excision of damaged nucleotides by enzymes discussed above). The action of Pol μ and Pol *λ* thus further extends parallels between NHEJ and BER/SSBR, and indeed Pol *λ* has roles in both [97-99], similar to APTX, PNKP and APLF.

## Trial and error

NHEJ can use enzymes that specifically and precisely resolve many of the end structures expected to interfere with ligation of DSB ends. This means some fraction of NHEJ can proceed by an ordered, three-step strategy (excision, replacement, ligation; Figure 4) essentially equivalent to that used by base excision repair. However, such a strategy will not always suffice for DSBs. Some end structures do not appear to be readily resolved by the available enzymes (e.g. reduced abasic sites; [27,29]). Additionally, it is not yet clear how well NHEJ can identify the enzyme appropriate to a given context; this may be particularly challenging at ends associated with densely-clustered damage. A fraction of NHEJ could thus proceed by “trial and error”. In this strategy, ends would be subject to sequential ligation attempts (Figure 5, step i, “trial”) and end processing (Figure 5, step ii, “error”), with these steps repeated until a substrate for ligation is generated. We suggest that efficient resolution by this strategy implies a need for regulated transition between steps.

This strategy employs processing factors that are more likely to be endonucleases that target secondary structures (single stranded overhangs, flaps, hairpins) and exonucleases, including Artemis [100], MRN [101], Werner’s syndrome protein [102], APLF [61], and Metnase [103]. Substrate specificities of these nucleases can be overlapping, and are generally less precisely targeted than the damage specific activities discussed above. Importantly, the latter characteristic allows them to aid in resolution of a wider variety of blocking lesions (more flexible), but the ensuing products are typically associated with greater deletion of DNA flanking the break site and are more heterogeneous. Initial overhang sequence complementarity will thus be reduced or lost, necessitating additional rounds of processing for ligation.

### Artemis

Artemis has been associated with 5^′^>3^′^ exonuclease activity, but can be primarily linked to NHEJ through an important role for its structure-specific endonuclease activity [100]. The latter is mediated by a metallo β lactamase associated with CPSF Artemis SNM1/PSO2 domain [104-106]. Nuclease activity is dependent on the additional presence of DNA-PKcs at ends [100], as well as DNA-PKcs autophosphorylation [107]. Artemis’s C terminus is also phosphorylated by both DNA-PKcs and the related Ataxia Telangiectasia Mutated (ATM) kinase [106,108-112], and this may further help regulate Artemis activity. Most notably, Artemis is required for opening hairpins at broken ends. Such structures are critical intermediates in the assembly of antigen specific receptors by V(D)J recombination, consequently loss of Artemis function results in severe immunodeficiency [100,113]. Loss of Artemis also confers cellular sensitivity to IR [113-115], suggestive of a role for this nuclease in resolving complex end structures expected from IR-induced breaks. Consistent with this, Artemis generally cleaves at ssDNA/dsDNA transitions, and thus can remove extended (>4 nucleotide) ssDNA overhangs and flaps [100,109], as well as overhangs with ligation blocking nucleotide damage [116]. Notably, products of Artemis nuclease activity are heterogenous, with sites of cleavage often distributed over a 3-6 nucleotide range [100,109,116,117]. For example, Artemis, like Tdp1, can excise 3^′^-phosphoglycolate termini *in vitro* but Artemis typically deletes a variable number of nucleotides in addition to the 3^′^-phosphoglycolate residue [116]. Both the increased deletion and deletion heterogeneity associated with Artemis activity will more frequently necessitate additional rounds of processing before ends can be ligated, relative to the damage specific activities described in the previous section [87].

### Werners syndrome protein

Werners syndrome is associated with progeria [118] and mild cellular sensitivity to ionizing radiation [119]. The Werners syndrome gene product (WRN) has both 3^′^>5^′^ exonuclease and helicase activity [120-122], and associates with NHEJ core factors Ku [102,123] and X4-LIV [124]. WRN activity is further regulated by DNA-PKcs [119,125], and can cooperate with these NHEJ factors to promote ligation *in vitro* after degradation of non-complementary overhangs [124]. Notably, WRN can degrade through oxidative damage in the presence of Ku in some contexts [126,127], possibly promoting NHEJ at ends where the density of break-associated damage is too high for damage specific resolutions.

### Mre11/Rad50/Nbs1

The Mre11/Rad50/Nbs1 (MRN) complex (or Mre11/Rad50/Xrs2 in *S. cerevisae*) is required for efficient sensing of double strand breaks and helps bridge broken ends together (reviewed in [128]). Similar to Artemis, MRN also has 3^′^>5^′^ exonuclease and single strand specific endonuclease activities (including hairpin opening activity) [101,129], but while MRN has an important role in mammalian NHEJ [130-132] this role is only partly reliant on MRN’s nuclease activity [132]. Notably, MRN nuclease activity is essential for removal of Spo11 covalently adducted to 5^′^ termini (a substrate analogous to aborted Topoisomerase II complexes) [79,133]. However, activity of the MRN and CtIP complex on this substrate precedes a more processive resection of 5^′^ ends (reviewed in [134]) that primarily channels these breaks to repair by the homologous recombination pathway. Indeed, MRN/CtIP may perform this function whenever end structures cannot be resolved by any of the NHEJ strategies discussed (Figure 5 step iii), allowing these ends to be resolved instead by either alternate end joining or homologous recombination. Consistent with this idea, MRN’s nuclease activity has been implicated in release of Ku from DNA ends [135], thereby precluding further futile NHEJ attempts.

## Concluding remarks: is it better to be lucky than good?

Complex end structures are diverse, explaining why NHEJ employs three distinguishable strategies (Figure 2) and a host of different processing activities (Table 1). Moreover, processing activities have varying degrees of substrate specificity, allowing NHEJ to balance precision with flexibility during end processing. When processing is required, these steps are appropriately ordered and coupled to the ligation step within a multiprotein machine. NHEJ is therefore indispensible for efficient resolution of complex end structures.

Ideally, NHEJ chooses a resolution strategy and processing factor in a manner that optimizes the efficiency and fidelity of product. With respect to strategy, there may be a means for sensing the extent of damage first and specifically choosing the appropriate strategy (Figure 2). Alternatively, strategies may be employed hierarchically, starting with damage tolerance, followed by damage specific end cleaning, followed by trial and error. Additionally, the choice of processing factor may be determined only by its affinity for a specific substrate, or might additionally be regulated by access to its substrate.

Accumulating evidence implicates DNA-PKcs kinase activity as the primary factor that could determine both choice of strategy and processing factor. DNA-PKcs kinase activity is dependent on end context, both in terms of whether a pair of ends can be aligned together [136,137], but also as a reflection of differences in end structure [138-140]. Additionally (as noted above), DNA-PKcs-mediated phosphorylation of Artemis [107-112], PNKP [51,52], Tdp1 [65], WRN [119,125], and XLF-X4-LIV [31] can affect the activity of these proteins. However, DNA-PKcs itself is probably the most relevant target (i.e., autophosphorylation), as there are in excess of 30 different sites (reviewed in [141]) that together may be sufficient for a “code,” where phosphorylation of different patches has distinguishable effects on end access [142-144].

Events in the resolution of complex ends by NHEJ can thus be, to some extent, left to chance, but can also be precisely scripted. So, is it better to be lucky than good? Why not both?

## Competing interests

The authors declare no competing interests.

## Authors’ contributions

NTS, CAW, and DAR wrote the manuscript, and N.T.S. assembled the figures. All authors read and approved the final manuscript.
